# Investigating Novel Syntheses of a Series of Unique Hybrid PLGA-Chitosan Polymers for Potential Therapeutic Delivery Applications

**DOI:** 10.3390/polym12040823

**Published:** 2020-04-04

**Authors:** Jason Thomas Duskey, Cecilia Baraldi, Maria Cristina Gamberini, Ilaria Ottonelli, Federica Da Ros, Giovanni Tosi, Flavio Forni, Maria Angela Vandelli, Barbara Ruozi

**Affiliations:** 1 Te.Far.T.I.-Nanotech Lab, Department of Life Sciences, University of Modena and Reggio Emilia, 41121 Modena, Italy; jasonthomas.duskey@unimore.it (J.T.D.); ilaria.ottonelli@unimore.it (I.O.); federica.daros93@gmail.com (F.D.R.); gtosi@unimore.it (G.T.); flavio.forni@unimore.it (F.F.); mariaangela.vandelli@unimore.it (M.A.V.); 2 Umberto Veronesi Foundation, 20121 Milano, Italy; 3 Department of Life Sciences, University of Modena and Reggio Emilia, 41121 Modena, Italy; cecilia.baraldi@unimore.it (C.B.); mariacristina.gamberini@unimore.it (M.C.G.); 4 Clinical and Experimental Medicine PhD Program, University of Modena and Reggio Emilia, 41121 Modena, Italy

**Keywords:** PLGA, chitosan, hybrid polymers, chitosan-PLGA polymer, NMR, DSC, FT-IR

## Abstract

Discovering new materials to aid in the therapeutic delivery of drugs is in high demand. PLGA, a FDA approved polymer, is well known in the literature to form films or nanoparticles that can load, protect, and deliver drug molecules; however, its incompatibility with certain drugs (due to hydrophilicity or charge repulsion interactions) limits its use. Combining PLGA or other polymers such as polycaprolactone with other safe and positively-charged molecules, such as chitosan, has been sought after to make hybrid systems that are more flexible in terms of loading ability, but often the reactions for polymer coupling use harsh conditions, films, unpurified products, or create a single unoptimized product. In this work, we aimed to investigate possible innovative improvements regarding two synthetic procedures. Two methods were attempted and analytically compared using nuclear magnetic resonance (NMR), fourier-transform infrared spectroscopy (FT-IR), and dynamic scanning calorimetry (DSC) to furnish pure, homogenous, and tunable PLGA-chitosan hybrid polymers. These were fully characterized by analytical methods. A series of hybrids was produced that could be used to increase the suitability of PLGA with previously non-compatible drug molecules.

## 1. Introduction

The discovery of effective therapeutic drugs is becoming increasingly difficult as seen by the drastic decline of new therapeutics accepted for public use each year. This is seen even with advances in structure activity relationship (SAR) studies [1], computer simulations of target structures (specific binding sequences and shape elucidation) [2], and high throughput screening methodology [3]. Novel surfaces and delivery nanosystems have taken the spotlight as the leading hope to advance new drugs from research into and beyond clinical studies by overcoming factors such as: lack of solubility, poor stability, poor biodistribution, immune response activation, off-target affects, and poor accumulation at the target site. Polymeric and lipid formulations have been taken advantage of to create fine-tuned systems to include targeting [4,5,6], triggerable activation (heat, light, reactive oxygen species (ROS), pH) [7,8,9], and varied uptake mechanisms to deliver pharmaceutics against numerous diseases [10,11,12].

In this respect, poly(lactic-co-glycolic acid) (PLGA) is of high interest due to the fact that it is: (1) FDA approved; (2) chemico-physically tunable to match biodistribution or loading needs; (3) capable of producing both nanosystems or polymeric scaffolds; (4) chemically modifiable to include stealthing moieties (polyethylene glycol, PEG) and/or targeting ligands. All of these aspects have been widely exploited in production of PLGA nanoparticles (NPs) for the possible cure of a plethora of diseases [13,14,15,16,17,18].

While PLGA NPs display many advantages in drug formulation, in comparison with cationic bio/polymers, they can suffer poor encapsulation efficiency when loading negatively-charged molecules. For example, while cationic bio/polymers (i.e., chitosan, cationic lipids, poly-ethylenimine, etc.) [19] can ionically bind negatively-charged DNA and form polyplexes, repulsion between the negatively-charged gene material and PLGA leads to negligible loading efficiencies. In this view, production of a co-polymer including chemical features needed for controlled release, absence of charge repulsion, and stable loading within the protective hybrid polymer assembly could be the correct answer to these limitations.

Previously, attempts to overcome these limitations were investigated in various ways. First, by surface engineering negatively-charged NPs (such as PLGA) with cationic molecules in order to allow DNA absorption onto the surface [20,21,22,23]. While this approach could improve theoretical loading of gene material or other positively-charged molecules onto polymeric NPs, the stability of the exposed drugs in a biological environment and control of their release are still lacking. Secondly, a synthesis of chitosan on a PLGA film for adsorption of hydrophilic molecules of chitosan for protein loading [24]. While in this study loading was improved, the reaction was only monitored based on time and the film remained intact throughout all analysis and the presence of absorbed but not reacted chitosan could be present. By creating a controlled synthesis of hybrid polymers, it would be possible to include improved encapsulation of drugs into PLGA assemblies, improving encapsulation of the molecule as well as protecting it within the structure from desorption in the blood and degradation. Furthermore, systematically synthesizing series of hybrid polymers could allow for tunability to include controlled release kinetics and degradation kinetics of the molecules as well.

Therefore, in this research we attempt two different synthetic methods to create a pure hybrid PLGA-chitosan polymer series: solid phase synthesis on a film (adapted from Li et al. [24]), or in solution chemical reaction (adopted from a reaction to react chitosan to polycaprolactone [25]). This will allow for the synthesize of a unique series of PLGA-chitosan hybrid polymers with tailored and tunable physico-chemical characteristics that could be used to expand the use of PLGA delivery systems of currently incompatible drugs or environments and in a variety of drug delivery assemblies to treat a larger range of disease states.

## 2. Materials and Methods

### 2.1. Materials

Poly (d,l-lactide-co-glycolide) acid [PLGA RG-503H 50:50, inherent viscosity in 0.1% (*w*/*v*) chloroform (CHCl_3_) at 25 °C = 0.38 dLg^−1^] was used as received from the manufacturer (Boehringer-Ingelheim, Ingelheim am Rhein, Germany). According to the experimental titration results of the carboxylic end of the polymers (4.94 mg potassium hydroxide (KOH)/g polymer) the molecular weight of RG-503H was calculated to be 11,000 Da. Low Molecular Weight chitosan, (mw 14,000) was purchased from Sigma Aldrich (Sigma Aldrich, Milano, Italy). All the solvents were of analytical grade, and all other chemicals and media were used as received from the manufacturers, and unless otherwise indicated, obtained from Sigma-Aldrich (Sigma Aldrich, Milano, Italy).

### 2.2. Solid Phase Synthesis of PLGA-Chitosan Co-Polymer

**Scheme 1:**
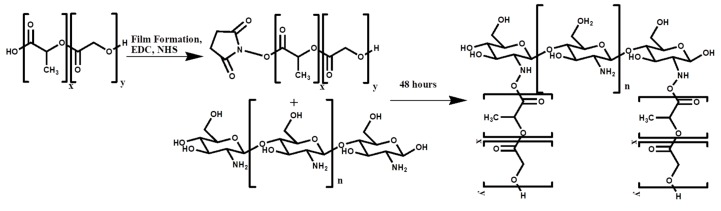
Solid phase reaction of PLGA film with chitosan.

The solid phase reaction of PLGA and chitosan was performed following the method of Ai.D. Li et al. with minor modifications (Scheme 1) [24]. Briefly, a PLGA solution (50 mg) was weighed into a round bottom flask and solubilized in 5 mL dicloromethane (DCM) and dried by rotary evaporation to create a thin film. The film was then washed for 1 h with 5 mL 6 *w*/*v*% NaOH (sodium hydroxide). This solution was discarded and the film was gently washed three times with 10 mL dilute HCl (hydrochloric acid 10%) followed by three more times with distilled water. The film was then completely covered in a solution containing N-Hydroxysuccinimide (NHS, 10 mgmL^−1^) and 1-Ethyl-3-(3-dimethylaminopropyl)carbodiimide (EDC, 10 mgmL^−1^) and reacted for another 6 h at room temperature in order to activate the acid group of PLGA with the NHS ester to promote the amide coupling with the amine of chitosan. This solution was discarded and the film was ultimately covered by a solution of 80 mL (reaction in round bottom flask) chitosan of 25 mgmL^−1^ (pH 3.5). Remarkably, to achieve this pH value in which the chitosan becomes more soluble with decreasing pH it becomes highly viscous, HCl (1N) was added dropwise and stirred vigorously for several minutes between each additional drop. Therefore, rigorous stirring for several minutes is needed in order to ensure the added HCl is dispersed uniformly throughout the solution and to avoid pockets of extreme acidity. After reacting for 48 h, the chitosan solution became much more transparent and less dense. The same procedure up to this point was also performed on a film on the surface of a glass petri dish (diameter 10 cm) with the following changes: the PLGA (150 mg) in 9 mL DCM evenly dispersed over the surface of the petri dish was left to evaporate at room temperature under a chemical hood overnight instead of on a rotary evaporator. The volumes required to cover the film with ~1 cm of each solution were decreased: NaOH 15 mL, EDC 15 mL (10 mgmL^−1^), NHS (15 mL 15 mL (10 mgmL^−1^), chitosan 15 mL (25 mgmL^−1^, pH 3.5). This decrease in volume was possible because unlike in the round bottom flask where a large volume is needed to fill in the 3D spherical space, on a flat surface the volume needed to cover the film is a much smaller cylindrical cross-section of the round bottom flask (1 cm thick cylinder). All material from the round bottom or petri dish was poured into a separation funnel and the reaction vessel was washed 3 × with water followed by 3 × with DCM (10 mL each), in order to remove products and starting material that are soluble in aqueous or organic solvents, and added to the separatory funnel. After allowing the extraction to separate for 1 h at room temperature in the separation funnel, three distinct layers formed during separation: a clear DCM layer, a middle white emulsion, and the yellow chitosan solution. The three layers were separated into separate containers and lyophilized to calculate a percent yield and further characterization.

### 2.3. Characterization Protocols of PLGA-Chitosan Co-Polymer in Solid-Phase.

Characterization of chitosan-PLGA co-polymer was achieved by analysis in FTIR, and by NMR. The FT-IR spectra were recorded by a Vertex 70 (Bruker Optics, Ettlingen, Germany) FT-IR spectrophotometer, equipped with a deuterium triglycine sulphate (DTGS) detector (Bruker Optics, Ettlingen, Germany). Setting parameters are: resolution 4 cm^−1^; apodization weak. The spectral range was 4000–600 cm^−1^ with 32 scans for each spectrum. The ATR spectra were recorded using the Golden-Gate accessory (Golden Gate™ Single Reflection Diamond ATR Series MkII).

1H NMR and 13C samples of the solid phase reactions were run on a Bruker 600 mHz NMR (Bruker, Milano, Italy). Simply, 4 mg of sample were dissolved in deuterated water with 1% v/v deuterated acetic acid added (Chitosan, 700 uL), or deuterated dimethylsulfoxide (DMSO) (PLGA-chitosan product, 700 uL), scanned 40 (1H) or 3000 (13C) times and analyzed by Bruker Top Spin software (Bruker, Milano, Italy).

### 2.4. Reaction of Chitosan and PLGA in Solution

**Scheme 2:**
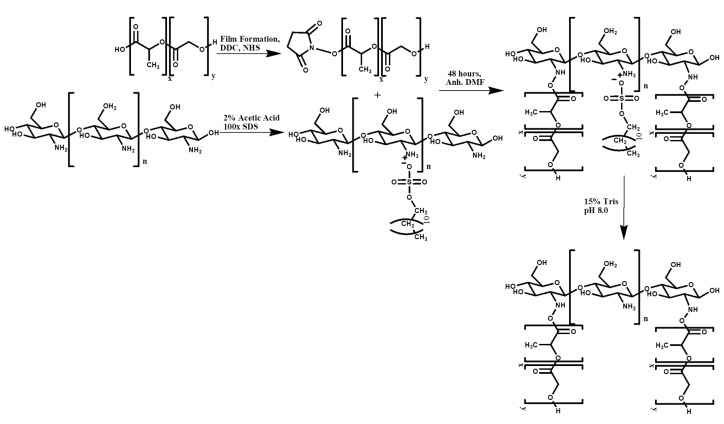
Solution Phase Reaction using DMF soluble chitosan-SDS salt.

To perform the conjugation between chitosan and PLGA in organic solution, an organic soluble SDS-chitosan salt was formed (Scheme 2). In particular, adapting a protocol published by Cai et al. [25], a solution of chitosan (200 mg) in 2% *v*/*v*) acetic acid was precipitated with SDS (Sodium dodecyl sulfate 560 mg) in a rapport of 1:100 for 2 h. The reaction was centrifuged for 10 min at 10,000 rpm in an ALC PK121 multispeed centrifuge (Concordia, Modena, Italy), the supernatant was discarded, and the precipitate was dried in a desiccator under negative pressure overnight. Simultaneously, PLGA was activated for reaction with chitosan by means of NHS-DDC technology. The covalent binding between the carboxy terminus of the polymer PLGA RG503H and the terminal amine of the peptide has been formed by standard methods, namely the activation of the carboxy group of PLGA by means of an ester with N-hydroxysuccinimide in the presence of dicyclohexylcarbodiimide, and the subsequent formation of an amidic linkage with the N-terminus of the unprotected peptide. Thus, to a solution of PLGA RG503H (1.00 g, 88 μmol) in anhydrous dioxane (5 mL), DCC (dicyclohexylcarbodiimide. 19.0 mg, 93 μmol) and N-hydroxysuccinimide (NHS, 11.0 mg, 93 μmol) were added, and the mixture was stirred for 4 h at 20 °C. After, the dicyclohexylurea was filtered away and the solution was decanted into cold anhydrous diethyl ether. The insoluble polymer was collected and purified by dissolution in DCM, followed by precipitation by the addition of anhydrous diethyl ether, then dried under reduced pressure. The content of NHS groups reacted with PLGA RG503H was determined by 1H-NMR spectroscopy (DPX 200; Bruker, Rheinstetten, Germany) in DMSO-d6, from the relative peak area of the multiplet at 2.95 ppm and of the multiplet at 1.80–1.60 ppm, corresponding to the protons of the N-succinimide and those of the methyl groups of the polymer, respectively, and resulted to be 49 μmol NHS/g of polymer. After having obtained both polymers, a fixed amount (50 mg) of the chitosan salt was then solubilized in anhydrous dimethylformamide (DMF, 10 mL) and reacted with different amounts (10, 50, or 240 mg) of activated PLGA-NHS (corresponding to ratio 1:5, 1:1, 5:1 chitosan: PLGA, respectively) and reacted for 48 h.

All the products were purified and isolated by means of centrifugation at 12,000 rpm for 10 min to remove any precipitated material during the reaction. The supernatant was then dried by rotoevaporation to yield a white/yellow powder containing the PLGA-chitosan/SDS salt conjugate. The SDS was then removed from the conjugate, which led to a precipitation of the final product by incubation in 50 mL 15% TRIS pH 8.0 for 48 h. The precipitate was then centrifuged at 12,000 rpm with an ALC PK121 multispeed centrifuge and the supernatant was decanted away. The final product was then dried by lyophilization and stored in a desiccator at room temperature until analysis. Solubility of the samples was tested by weighing 1 mg of each product and testing its ability to dissolve in DMSO or acetic acid (2% *v*/*v*) of concentrations of 200 ugmL^−1^.

### 2.5. Characterization Protocols of PLGA-Chitosan Co-Polymer in Solution

FTIR was described as previously described. The FT-IR spectra were recorded by a Vertex 70 (Bruker Optics, Ettlingen, Germany) FT-IR spectrophotometer, equipped with a deuterium triglycine sulphate (DTGS) detector. Setting parameters are: resolution 4 cm^−1^; apodization weak. The spectral range was 4000–600 cm^−1^ with 32 scans for each spectrum. The ATR spectra were recorded using the Golden-Gate accessory (Golden Gate™ Single Reflection Diamond ATR Series MkII).

After purification, NMR spectra were acquired at 300 K using an AVANCE III HD 600 Bruker spectrometer, equipped with a 2.5 mm H/X CPMAS probe operating at 600.13 and 150.90 MHz for 1H and 13C, respectively (Bruker, Milano, Italy). Samples were packed into 2.5 mm zirconia rotors and spun at the magic angle. 13C NMR spectra were obtained using a standard pulse sequence for cross polarization (CP), at 16 kHz magic angle spinning (MAS) rate. The relevant acquisition parameters for CP-MAS 13C NMR spectra were: 45 kHz spectral width, 10 s relaxation delay, 2.5 μs 1H 90° pulse, 62.5 kHz radio frequency field strength for Hartmann−Hahn match, 2k data points, and 2k scans. All chemical shifts were referenced by adjusting the spectrometer field to the value corresponding to 38.48 ppm chemical shift for the deshielded line of the adamantane 13C NMR spectrum.

Dynamic scanning Calorimetry of DSC was performed on a Netzsch Phox DSC 200 PC using the Netzsch Proteus analysis software (NETZSCH-Gerätebau GmbH, Selb, Germany). Samples were precisely weighed between 2–4 mg each into NETZSCH DSC-crucibles (Al; 25 uL) and sealed with their appropriate lids. An empty crucible was used as the reference sample. Samples were analyzed with the following thermometric gradient: 2 min isothermal gradient to standardize the starting point at 15 °C, 15–320 °C over 38 min increasing at 10 °C per minute, with a 2 min isothermal section.

## 3. Results

The most direct method of conjugating PLGA and chitosan is an amid bond formation between the amine on chitosan and the carboxylic acid of PLGA (Scheme 1). Functionally however, this reaction is complicated due to the extreme difference in solubility between the two molecules. Previous attempts reacted chitosan in solution with a PLGA film to create a positively-charged surface aiming to create nanofibers without the need for purification [24]. Another researcher produced the hybrid PLGA-chitosan polymer for the creation of nanoparticles, but it required harsh conditions (concentrated nitric acid) [26]. Therefore, to create a pure, reproducible, and controllable hybrid polymer that could be used in solution for NP formation, a reaction was performed under milder conditions on a PLGA film created by evaporating PLGA on a surface activating it with EDC and NHS and reacting it with a large excess of chitosan. After 48 h, the chitosan solution was removed and the product was purified in a biphasic solution of 0.1% acetic acid (PLGA-chitosan product) and DCM (non-reacted PLGA). Initial reactions were performed in a round bottom flask; however, to make the reaction greener by decreasing the ratio of surface area:volume (to decrease amount of solvent and reactants needed to cover the PLGA film), reactions were performed in a flat petri dish. This simple change not only decreased reaction volume (80 mL to 10 mL), but it also increased the % yield from ~25% to 50%.

**Figure 1:**
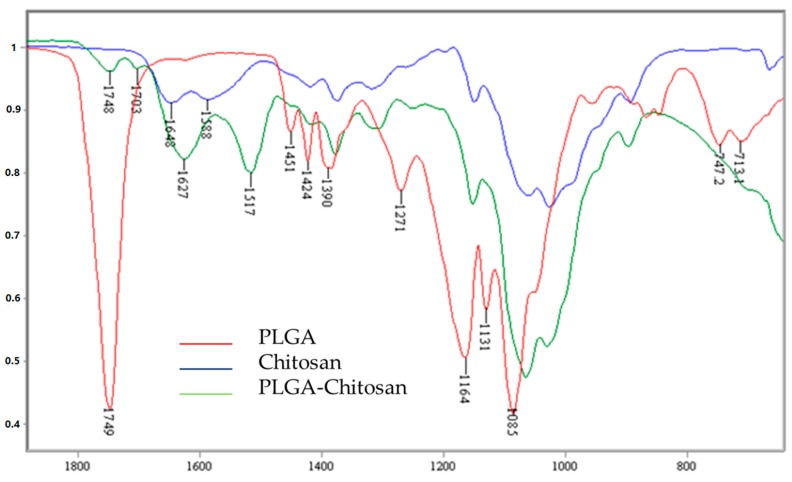
FT-IR/ATR spectra of unmodified poly lactic-co-glycolic acid (**red**), unmodified chitosan (**blue**), and PLGA-chitosan product (**green**).

Characterization of the product was performed by FTIR spectroscopy (a common technique for investigating interactions between polymers) and NMR. General FTIR points of interest for the reaction arise in the broad band between 3450 and 3200 cm^−1^ (νOH + νNH) and two weak peaks at 2940 cm^−1^ and 2890 cm^−1^ (νCH2) (Figure S1, top panel). More critical for the identification of the conjugation of PLGA to chitosan are the major characteristic absorption bands at around 1648 and 1588 cm^−1^, corresponding to amide I (νC=O) and amide II (δNH + νCN) of the residual N-acetyl groups. Under the band centered at 1585 cm^−1^, the contribution of δNH2 is also hidden, which overlaps the amide II peak [27]. Pure PLGA exhibits the strong characteristic adsorption peaks at 1170 and 1090 cm^−1^ (νCOC, ether), 1130 cm^−1^ (ρCH3), 1452, 1390, and 745 cm^−1^ (δCH), and peaks 3020 and 2930 cm^−1^, which were attributed to νCH2 from glycolic acid portion, and νCH3 from the lactic acid portion. The most notable peak to discern the presence of PLGA arises at 1749 cm^−1^ (νC=O, ester) (Figure S1, bottom panel) [28]. FTIR analysis of the product showed that the mild acetic acid conditions did not result in the covalent linkage between PLGA and chitosan (Figure 1). While a shift in the amid bonds at 1648 and 1588 cm^−1^ were observed, the new peaks did not correspond to further amid bond creation, but instead showed the resemblance of the formation of a chitosan salt [29] with bands further downfield at 1627 and 1517 cm^−1^. Also, only a small emergence of a peak indicating the presence of the C=O of PLGA at 1748 cm^−1^, but instead a peak at 1703 cm^−1^ indicating the appearance of an acid was observed.

**Figure 2:**
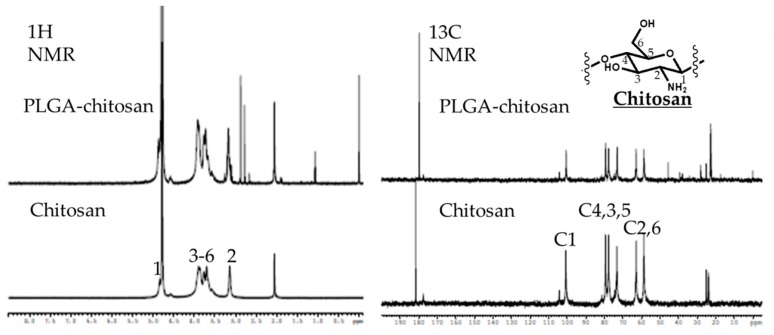
600 MHz NMR Proton (**left panel**) and Carbon (**Right Panel**) analysis. Numbers indicate the Carbons (C1-6) of the chitosan ring structure or their respective hydrogens as depicted in the structure (Top right).

NMR analysis confirms the poor reaction results. 1H NMR of the product and the chitosan control show little to no difference with the H2 peak at 3 ppm and the H3-6 peaks as a broad series of peaks at 3.5–4 ppm. These peaks corresponded to the literature precedence of the 13C NMR peaks of the main carbon ring at ~ C1 (100 ppm), C3-5 (73–85 ppm), and C2,6 (55–60 ppm). However, the normal peaks expected for the PLGA C=O (170 ppm) or CH3 (1.5 ppm and 15 ppm 13C) or its degradation products (glycolic and lactic acid) are not present (Figure 2). Only small fragment peaks that do not cross correlate upon 2D analysis (Figure S2). Degradation of the PLGA into small fragments during the reaction would explain the FTIR results showing the formation of a small acid peak. It also could explain the amid bond shifts to that of the salt formation as small negatively-charged acidic degradation products could lead to a salt formation with the free amine of chitosan.

While the reaction of PLGA and chitosan is found in the literature, it is often performed with harsh conditions on gels or without purification. Using a solid phase reaction with mild reaction conditions did not prove successful. This ruled out this method as a viable option to create a controlled series of hybrid polymer variants for further characterization; therefore, more stable and controllable methodology was pursued.

The mild reaction conditions led to a lack of product formation. To overcome this, an alternate method was adapted in which chitosan is precipitated as an SDS salt in order to improve its solubility in organic solutions (DMSO, choloroform, and DMF) [25].

This intermediate was then conjugated to PLGA-NHS in anhydrous DMF. To test the flexibility of the reaction, and to analyze the physical characteristics of various PLGA-chitosan hybrid polymers, a series of three reactions was performed: (1) an excess of chitosan in a 5:1 molar ration, (2) 1:1 chitosan: PLGA and (3) 1 chitosan: 5 PLGA (Table 1). The latter corresponds to an average of one PLGA being available for each sugar unit of chitosan. After reacting activated PLGA with chitosan-SDS for 48 h, the salt was dissociated in Tris 15% pH 8 for 48 h. The percent yield of the reactions increased proportionally with the increasing rapport of PLGA: chitosan in the reaction as shown in Table 1 (55%, 75%, and 82%). The solubility of the products also suggested an increased PLGA attachment due to the decreasing solubility in 2% acetic acid (*v*/*v*).

**Figure 3:**
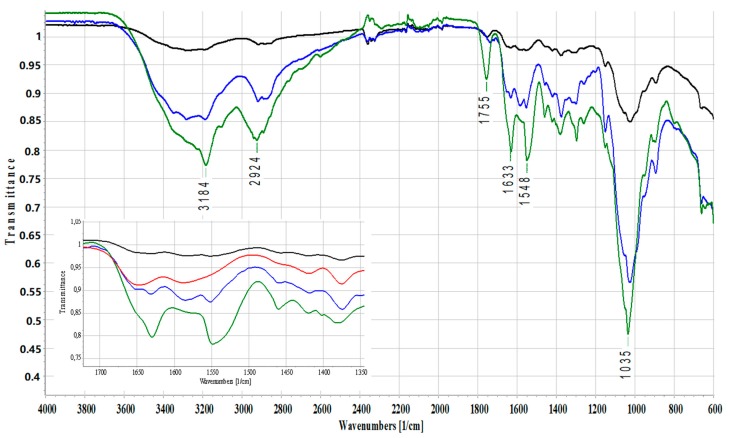
FTIR scan of PLGA-chitosan hybrid products of the reactions synthesized with different molar rapport of PLGA:chitosan of reaction (1) 1:5. black (2) 1:1. blue (3) 5:1. green, and (4) pure chitosan (red inset).

The conjugation of PLGA to chitosan again was analyzed by FTIR, and 13C NMR in solid state (due to the differences in solubility between the products). The PLGA and chitosan starting materials were identical to as described previously (Figure 1, Figure S1). In a PLGA concentration dependent manner, the progressive appearance and intensification of the band at 1755 cm^−1^, indicating the presence of PLGA (ester C=O stretching), can be observed (Figure 3). Unlike the solid-state reaction, bands indicating a chitosan salt formation were not observed (including the presence of the chitosan-SDS salt formation (1624 and 1523 cm^−1^)) (Figure S3). Instead, there was a clear and concentration dependent (based on initial PLGA amounts), shift indicating amid bond formation (Figure 3). This was confirmed by observing the amide I and II peaks (1650 and 1585 cm^−1^) shift to 1633 cm^−1^ (amide I) and 1548 cm^−1^ (amide II) (Figure 3). As compared with pure chitosan, the δNH contribution of the primary amine for the band at 1580 cm^−1^ decreases or even disappears, because of a change of primary amine in the chitosan chain into amide groups, as already attested in the literature [30]. The displacement of the band from 1586 cm^−1^ (chitosan) to 1549 cm^−1^ (PLGA-chitosan) suggests that the grafting reaction occurred mainly by the reaction between the −NH2 chitosan groups and −COOH PLGA groups. Furthermore, the band at 3184 cm^−1^ progressively increases, associated with NH stretching of the secondary amide (Figure 3 inset).

**Figure 4:**
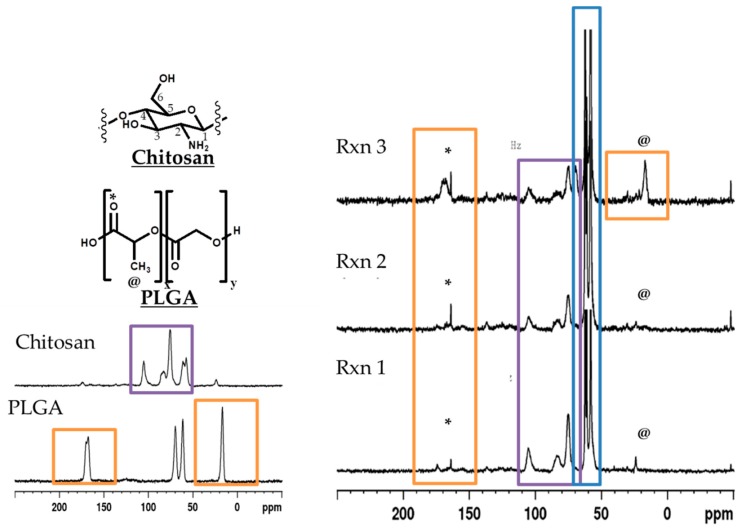
Solid state 13C NMR analysis with highlighted peaks of interest: chitosan (**purple**), PLGA (**orange**), TRIS salt (**blue**). Numbers indicate the Carbons (C1-6) of the chitosan ring structure or their respective hydrogens as depicted in the structure (Top left), * indicates the carbon of the PLGA carbonyl peak, and @ indicating the PLGA methyl group.

NMR analysis was used to support the FTIR findings (Figure 4). The solid-state NMR of pure chitosan showed the characteristic broad singlet at 100 ppm (carbon 1) along with two broad multiplet peaks between 50 pp, (carbon 2–5) and 90 ppm (carbon 6) in accordance with literature precedence (Figure 4 purple box) [31]. The pure PLGA exhibits the CH and CH2 peaks at 70 and 60 ppm, respectively, as well as the CH3 peak at 15 ppm and C=O peak at 170 ppm (Figure 4, orange boxes). In all reactions, the iconic peaks of the chitosan can be seen. In all three reactions, the PLGA peak corresponding to the CH2 is hidden under the chitosan (purple box) and residual TRIS salt (blue box) peaks from 50–75 ppm, but the emergence of the CH peak at 70 ppm is observed (Figure 4, Figure S4). More evident however; is that by increasing the initial amount of PLGA in the reaction, the peak corresponding to C=O at 170 ppm (indicated by a star) as well as that of the CH3 group (indicated by an @) are seen to directly increase in intensity (Figure 4 orange boxes). It is important to note that SDS, and Chitosan-SDS salt (peaks 20–40 ppm) are not present in any of the samples indicating full removal of the salt back to the original chitosan structure in the product (Figure S4, red box). NMR analysis showed constant and equal NMR spectra across multiple product samples indicating the homogeneity and controllability of each product.

**Figure 5:**
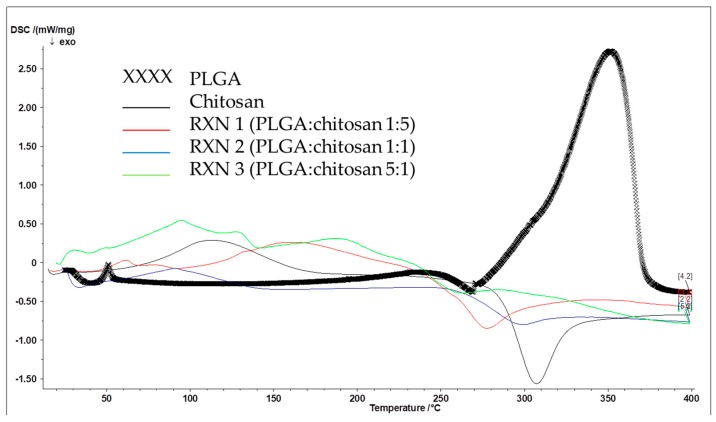
Dynamic scanning calorimetry analysi: PLGA (**black crosses**), chitosan (**black line**), and the three PLGA-chitosan reaction products based on PLGA:chitosan molar rapport: (1) 1:5 (**red**) (2) 1:1 (**blue**) and (3) 5:1 (**green**).

To further validate the conjugation of PLGA to chitosan, DSC analysis was performed (Figure 5). The transitional peak of PLGA was seen at 50 °C along with an endothermic transition during its degradation between 280–380 °C. The chitosan control shows the liberation of the water entrapped between the chitosan chains at 115 °C along with an exothermic transition at approximately 300 °C reasoned to be the degradation of the chitosan ring structures permitting 3-D rotation. Analysis of the polymer samples showed a shift in all transitional states dependent on the concentration of PLGA in the initial reaction solution. With increasing amounts of PLGA, the transitional phase at 50 °C disappeared due to the loss of the glass transition when bound to chitosan. In a physical mixture of PLGA and chitosan however this transition was still observed (Figure S5). Secondly, a shift to higher temperatures of the water loss from 120 °C to 150 °C in sample 3 (with the most PLGA) exhibiting numerous peaks in this range. The energy required to remove the water associated with the chitosan chains is increased by the increased encumbrance of PLGA. Finally, the disappearance of the transition peak at around 290 °C caused by the bulky PLGA sterically hindering the free rotation of the chitosan chains as well as cancellation of the endothermic (PLGA) and exothermic (Chitosan) energies were observed supporting the FTIR and NMR data of the presence of increasing amounts of PLGA chemically linked to the chitosan chain. To ensure these changes were not caused by the presence of the chitosan-sds salt, a sample was also analyzed showing none of the characteristics of the polymer products (Figure S5).

These three series of analysis not only demonstrate the formation of the hybrid polymer series using mild reaction conditions, but also show the versatility of the reaction in its ability to be stoichiometric controlled to create a uniform product unlike that seen by the solid-state reaction. The formation of the chitosan-SDS made it optimal for the reaction in organic solvents with PLGA. By varying the ration of PLGA in the reaction conditions from a 1:5 excess of chitosan, to 1:1, and finally to 5:1 excess of PLGA, it was possible to create a variation of hybrid polymers. The hybrid series was not only verified by the analytical characterizations, but also by the difference of solubility of the product. Controlling the reaction in a stoichiometric controlled manner to create such clean and reproducible product, hybrid polymers greatly increases the translatability and feasible uses of these polymers in drug delivery purposes.

## 4. Discussion and Conclusions

Finding new materials to stabilize molecules with poor stability, solubility, or biocompatibility properties is necessary to continue advancing new disease treatment methods with “critical” but non-compatible drugs. PLGA offers a very promising base material as it is FDA approved and has been used extensively to specifically target drugs to diseases as NPs or as site specific delivery agents inserted as a film but is limited in loading positively-charged molecules.

Creating new co-polymers in a constant and controlled manner offers an increasing utility of PLGA assemblies for a broader range of potential drug candidates in which it is currently non-compatible. To this end, two reaction methods were attempted to conjugate negatively-charged PLGA to the positively-charged chitosan to form a series of novel co-polymers. Previous works have attempted to make chitosan hybrid polymers using harsh reaction conditions (nitric acid), PLGA films, or in solution (to make polycaprolactone hybrid polymers) in a non-purified and uncontrollable manner. To truly benefit from these types of hybrid polymers, the reaction must be reproducible, controllable, create a series of pure homogenous products that can be selected dependent on the therapeutic need.

Data indicated that solid-phase synthesis using a PLGA film and mild reaction conditions was insufficient to create PLGA-chitosan hybrid polymers, but instead led to a salt formation with degradation products in solution. However, by utilizing an SDS salting out reaction to create a chitosan SDS intermediate that is soluble in organic solvents, a series of PLGA-chitosan co-polymers with different molar ratios were produced.

Remarkably, this reaction was able to furnish a unique series of pure and reproducible PLGA-chitosan hybrids with various molar rapport and solubilities. This controlled synthesis method makes these hybrids prime candidates for protection and delivery of a wide range of previously non-combatable drugs either as NPs formed through chitosan self-assembly techniques (for those still soluble in acidic solutions) or for the encapsulation in stable and non-toxic films for long-term controlled release (for those insoluble in biological solutions).

These preliminary results could pave the way to further advances in the application of PLGA-based nanotherapeutics, expanding the tunability of the core polymer structure to be better suited for a wider range of drugs candidates to be loaded, protected, and delivered to diseased cells improving their potency and efficacy.

## Figures and Tables

**Table 1:** Solution phase reaction conditions of PLGA and Chitosan-SDS.

Reaction	Chitosan-SDS (mg Chitosan)	PLGA-NHS (mg)	Recovery (%)	PLGA:Chitosan in Initial Reaction Solution (mol)	Solubility in Acetic Acid ^a^
1	50	10	55	1:5	soluble
2	50	50	75	1:1	semi soluble
3	50	240	82	5:1	Not soluble

a. 200 ug/mL 2% *v*/*v*.

## Supplementary Materials

The following are available online at https://www.mdpi.com/2073-4360/12/4/823/s1, Figure S1: Full FTIR Scan of PLGA and chitosan polymers, Figure S2: 2D NMR correlation analysis of the 1H and 13C PLGA-chitosan reaction product, Figure S3: FTIR analysis of chitosan control (Red), chitosan-SDS salt (green) and SDS salt (blue), Figure S4: Solid state 13C NMR analysis with highlighted peaks of interest: chitosan (purple), SDS (red), TRIS salt (blue), Figure S5: Dynamic scanning calorimetry analysis: chitosan-SDS salt (black line), chitosan: PLGA 1:1.5 physical mixture (green line).
