# Supplementary material for: Investigating Novel Syntheses of a Series of Unique Hybrid PLGA-Chitosan Polymers for Potential Therapeutic Delivery Applications
Source: Polymers (Basel). 2020 Apr 4;12(4):823. doi: 10.3390/polym12040823 (PMC7249265; doi:10.3390/polym12040823)
Supplement: Supplementary file 1 [file polymers-12-00823-s001.docx]

*Supplemental Material*


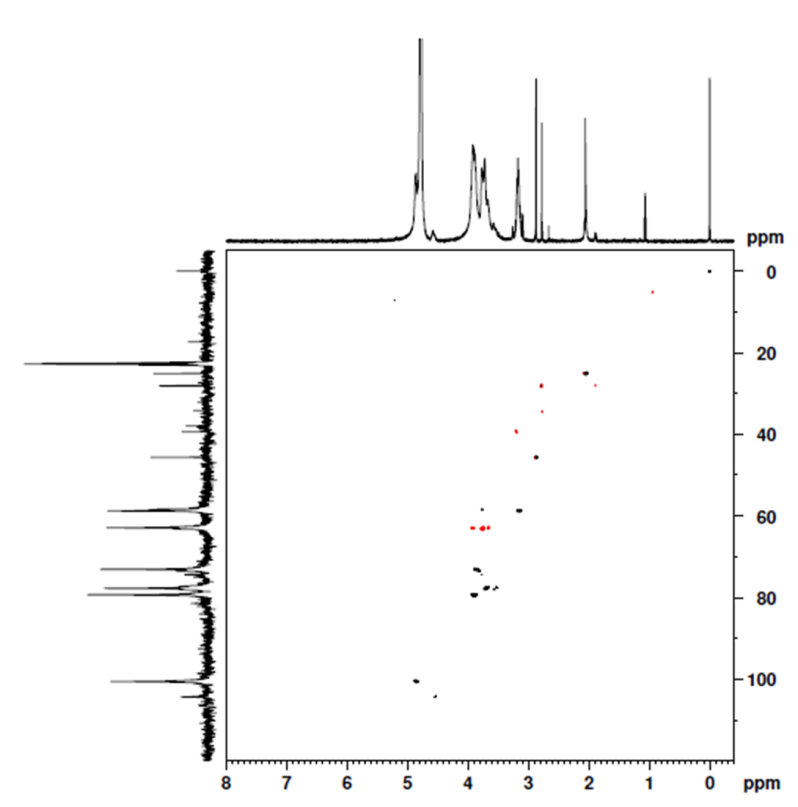

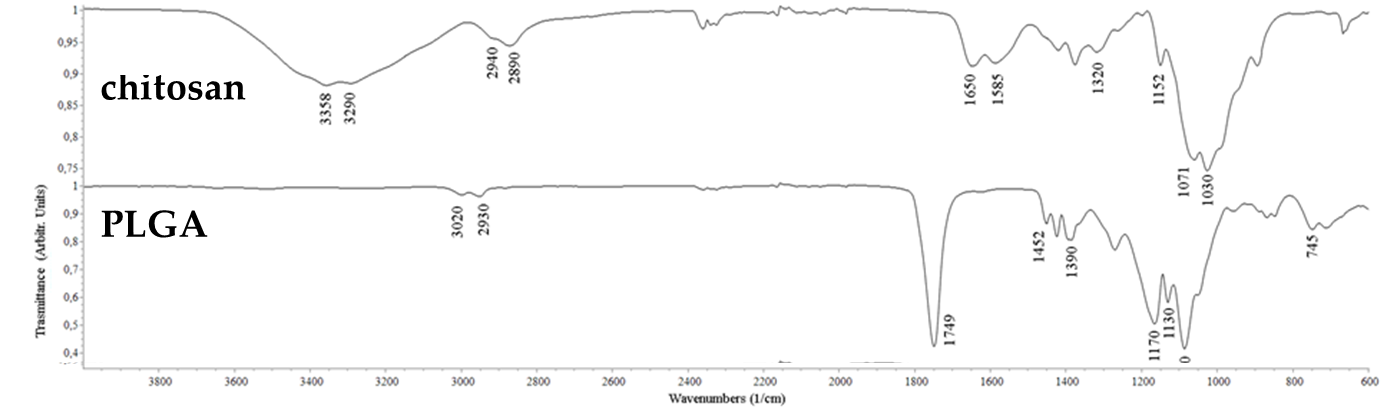


Figure S1: Full FTIR Scan of PLGA and chitosan polymers.

Figure S2: 2D NMR correlation analysis of the 1H and 13C PLGA-chitosan reaction product.


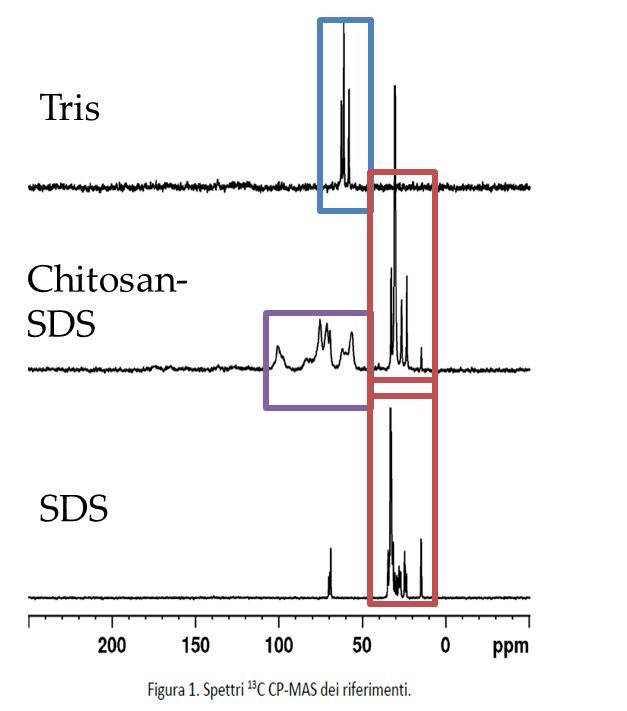

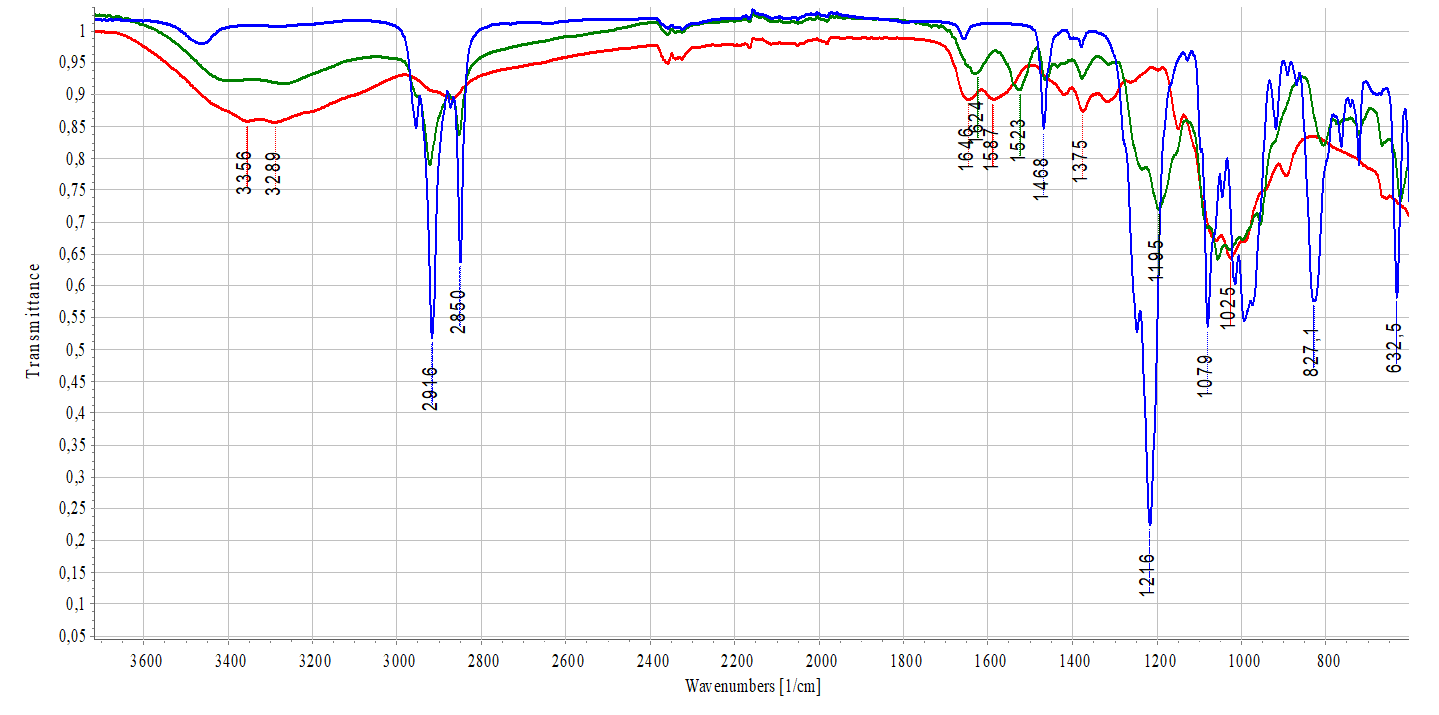


Figure S3: FTIR analysis of chitosan control (Red), chitosan-SDS salt (green) and SDS salt (blue).

Figure S4: Solid state 13C NMR analysis with highlighted peaks of interest: chitosan (purple), SDS (red), TRIS salt (blue).


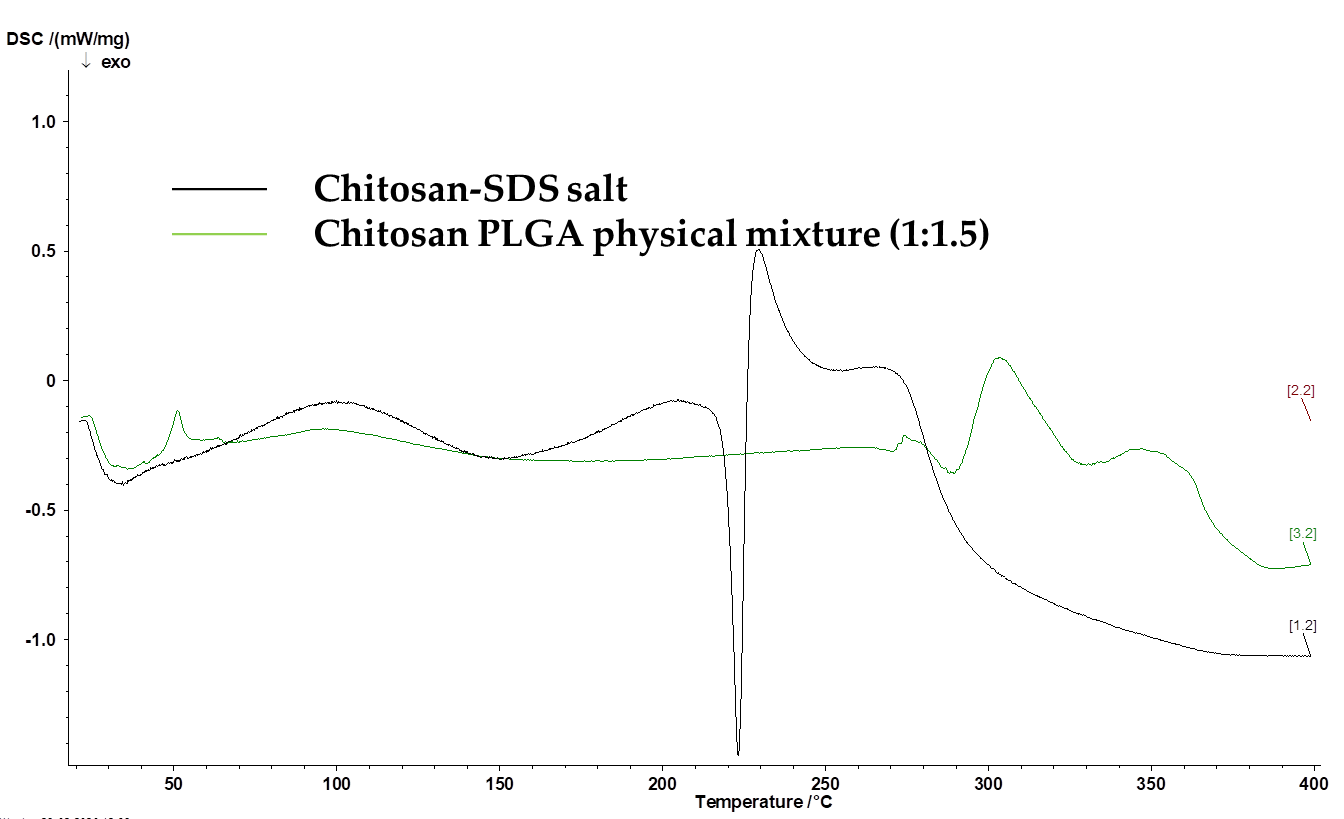


Figure S5: Dynamic scanning calorimetry analysis: chitosan-SDS salt (black line), chitosan : PLGA 1:1.5 physical mixture (green line).
